# More pilot trials could plan to use qualitative data: a meta-epidemiological study

**DOI:** 10.1186/s40814-020-00712-z

**Published:** 2020-10-29

**Authors:** Tejan Baldeh, Tonya MacDonald, Sarah Daisy Kosa, Daeria O. Lawson, Rosa Stalteri, Oluwatobi R. Olaiya, Ahlam Alotaibi, Lehana Thabane, Lawrence Mbuagbaw

**Affiliations:** 1 Department of Health Research Methods, Evidence and Impact, McMaster University Health Sciences Centre, McMaster University, 1280 Main Street West, Hamilton, ON L8N 4K1 Canada; 2 School of Midwifery, Laurentian University, Sudbury, ON Canada; 3 Toronto General Hospital, University Health Network, Toronto, ON Canada; 4 Michael G. DeGroote School of Medicine, McMaster University, Hamilton, ON Canada; 5 Department of Pediatrics, Princess Noura University, Riyadh, Saudi Arabia; 6 Biostatistics Unit, Father Sean O’Sullivan Research Centre, St Joseph’s Healthcare Hamilton, Hamilton, ON Canada; 7 Departments of Paediatrics and Anaesthesia, McMaster University, Hamilton, ON Canada; 8 Centre for Evaluation of Medicine, St Joseph’s Healthcare Hamilton, Hamilton, ON Canada; 9 Population Health Research Institute, Hamilton Health Sciences, Hamilton, ON Canada; 10 Centre for the Development of Best Practices in Health, Yaounde, Cameroon

**Keywords:** Pilot, Feasibility, Trials, Qualitative data, Protocols

## Abstract

**Background:**

Pilot trials often use quantitative data such as recruitment rate and retention rate to inform the design and feasibility of a larger trial. However, qualitative data such as patient, healthcare provider, and research staff perceptions of an intervention may also provide insights for a larger trial.

**Methods:**

As part of a larger study investigating the reporting of progression criteria in pilot studies, we sought to determine how often pilot studies planned to use qualitative data to inform the design and feasibility of a larger trial and the factors associated with plans to use qualitative data. We searched for protocols of pilot studies of randomized trials in PubMed between 2013 and 2017.

**Results:**

We included 227 articles. Only 92 (40.5%; 95% confidence interval [CI] 34.1–47.2) reported plans to collect qualitative data. The factors associated with collecting qualitative data were large studies (defined as sample size ≥ 60; adjusted odds ratio [aOR] 2.77; 95% CI 1.47–5.23; *p* = 0.002) and studies from Europe (aOR 3.86; 95% CI 1.68–8.88; *p* = 0.001) compared to North America and the rest of the world. Pilot trials with pharmacological interventions were less likely to plan to collect qualitative data (aOR 0.20; 95% CI 0.07–0.58; *p* = 0.003).

**Conclusions:**

Qualitative data is not used enough in pilot trials. Large pilot trials, pilot trials from Europe, and pilot trials of non-pharmacological interventions are more likely to plan for qualitative data.

## Background

Feasibility studies are a category of study which aim to determine if a study should be done, can be done, and, if so, how it might be done [1]. Pilot studies are a type of feasibility study that maintains an element of study design (e.g. randomization), albeit on a smaller scale than what is intended for the full trial [1–3]. Data from pilot studies is of great value to researchers for identifying and correcting problems which might otherwise compromise acceptability and delivery of interventions in the full study. By correcting these issues before carrying out a larger study, researchers may reduce misuse of resources and inappropriate evaluation techniques in the larger trial [4–6].

Since pilot studies are based on the methods intended for a full-scale investigation, quantitative and qualitative methods can be used. Both methods have the potential to yield useful data in the context of a pilot study, and it is recommended by the United Kingdom Medical Research Council (MRC) that qualitative and quantitative methods be used concurrently with a pilot study or full trial [5]. For example, in 2018, Irish researchers evaluated the feasibility and acceptability of a sexual counselling intervention for patients undergoing cardiac rehabilitation [7]. An evaluation was performed by documenting recruitment and attrition rates, as well as recording feedback from interviews with staff and patients.

Qualitative methods are increasingly being used in pilot trials [8]. The reason qualitative methods were not used as much in prior years is not entirely clear but may have been because of a lack of guidance on the topic at the time or limited understanding of the value of qualitative methods among certain researchers. In the past, some researchers have expressed concern regarding the practicality, reliability, and generalizability of qualitative methods [9]. However, in recent years, there has been an increase in the number of resources available to researchers seeking to perform and publish pilot studies. In 2015, the journal *Pilot and Feasibility Studies* was created, which has acted as a platform to promote pilot and feasibility research [10]. There has also been a considerable amount of guidance published on the use of qualitative methods in pilot studies [11] and reporting of pilot studies [12–14].

Given the recent developments in the field, practices regarding the use of qualitative methods in pilot studies may have changed. More information on the collection and use of qualitative data in pilot studies may help researchers develop further guidance and improve the design of pilot studies as well as the larger investigations they inform. With the publication of pilot study protocols becoming more common in *Pilot and Feasibility Studies*, we have greater insight into the researchers’ intentions to use qualitative methods [13]. Therefore, the purpose of this study was to determine how often pilot trials are designed to collect qualitative data and the study characteristics associated with planning to collect qualitative data. Accordingly, we conducted a methodological review of published protocols for pilot randomized trials. This work was conducted as part of another methodological study investigating the use of progression criteria in pilot studies [15].

## Methods

### Data collection

We conducted a methodological review of protocols for pilot randomized trials. We applied the following search strategy in the PubMed database, including terms for three journals known to publish protocols and pilot studies: (BMJ Open [Journal] OR Pilot Feasibility Stud [Journal] OR Trials [Journal]) AND (Pilot [Title] OR Feasibility [Title] AND Protocol [Title]). We restricted our search to studies published between 01 January 2013 and 31 December 2017—a 5-year period—to keep the data manageable.

Two separate reviewers (SDK, DOL, RS, ORU, AA, or LM) screened each of the full texts of identified citations for eligibility. In order to meet eligibility, the studies had to be (1) published in *Pilot and Feasibility Studies (PAFS)*, *British Medical Journal (BMJ) Open*, or *Trials*; (2) a protocol for a pilot randomized trial; and (3) published between 2013 and 2017. Data were extracted from the eligible studies by one reviewer and verified by a second independent senior reviewer. Verification involved comparing the extracted data to the manuscript to identify errors or discrepancies. New data were extracted by the second reviewer only when there were discrepancies or errors.

Reviewers determined whether each included pilot trial had planned to collect qualitative data (including but not limited to in-depth focus groups, one-on-one interviews, qualitative surveys/questionnaires) and the population from which the data were to be retrieved (i.e. participants, staff, investigators, others). Data on the study characteristics (i.e. bibliographic information, country of origin, source of funding, study objectives and outcomes, intervention type (pharmacological vs. non-pharmacological), whether the study considered feasibility outcomes, sample size estimation, and adequacy of the justification for sample size) were extracted. These characteristics were selected because they have been found to be associated with reporting in other studies [16]. When the planned sample size was reported as a range of values, the median was taken. When different sample sizes were reported for the different participants (e.g. health workers, patients, caretakers), we used the sample size for those who would be randomized. To facilitate analysis, we categorized the studies as small (< 60) or large (≥ 60), based on the median sample size (60) across studies. The current guidance suggests that the sample size of a pilot trial should be based on a feasibility outcome and not clinical outcomes (intervention effect size) [17]. As such, using the intervention effect size to determine the sample size, stating that it was selected because other studies reported the same way, or providing no justification was deemed inadequate. The country in which the pilot was planned was also collected and categorized into three world regions (North America, Europe, and rest of the world) to facilitate analyses (other regions contributed very few studies).

All data were collected and managed using the Research Electronic Data Capture (REDCap) tool hosted at St Joseph’s Healthcare Hamilton (Ontario, Canada) [18].

### Data analysis

First, counts of the plans to collect qualitative data, as well as the study characteristics, were summarized descriptively in cross tabulations. Second, we used logistic regression to determine the relationships between the study characteristics and the odds of planning to collect qualitative data (categorized as yes/no). The covariates were entered as a block: journal (PAFS, BMJ Open, and Trials), year of publication (continuous), region (North America, Europe, or rest of the world), source of funding (industry or government/private), intervention type (pharmacological or non-pharmacological), and sample size (small [0–60], large [> 60]). These variables have been shown to be associated with reporting standards [16]. The goodness of fit of the model was assessed using the Hosmer-Lemeshow test (*α* = 0.05). Crude odds ratios (OR) and adjusted odds ratios (aOR), corresponding 95% confidence intervals (CI), and *p* values are reported. Data was analysed using IBM SPSS Statistics version 25 and WinPepi (PEPI-for-Windows) [19].

## Results

**Fig. 1:**
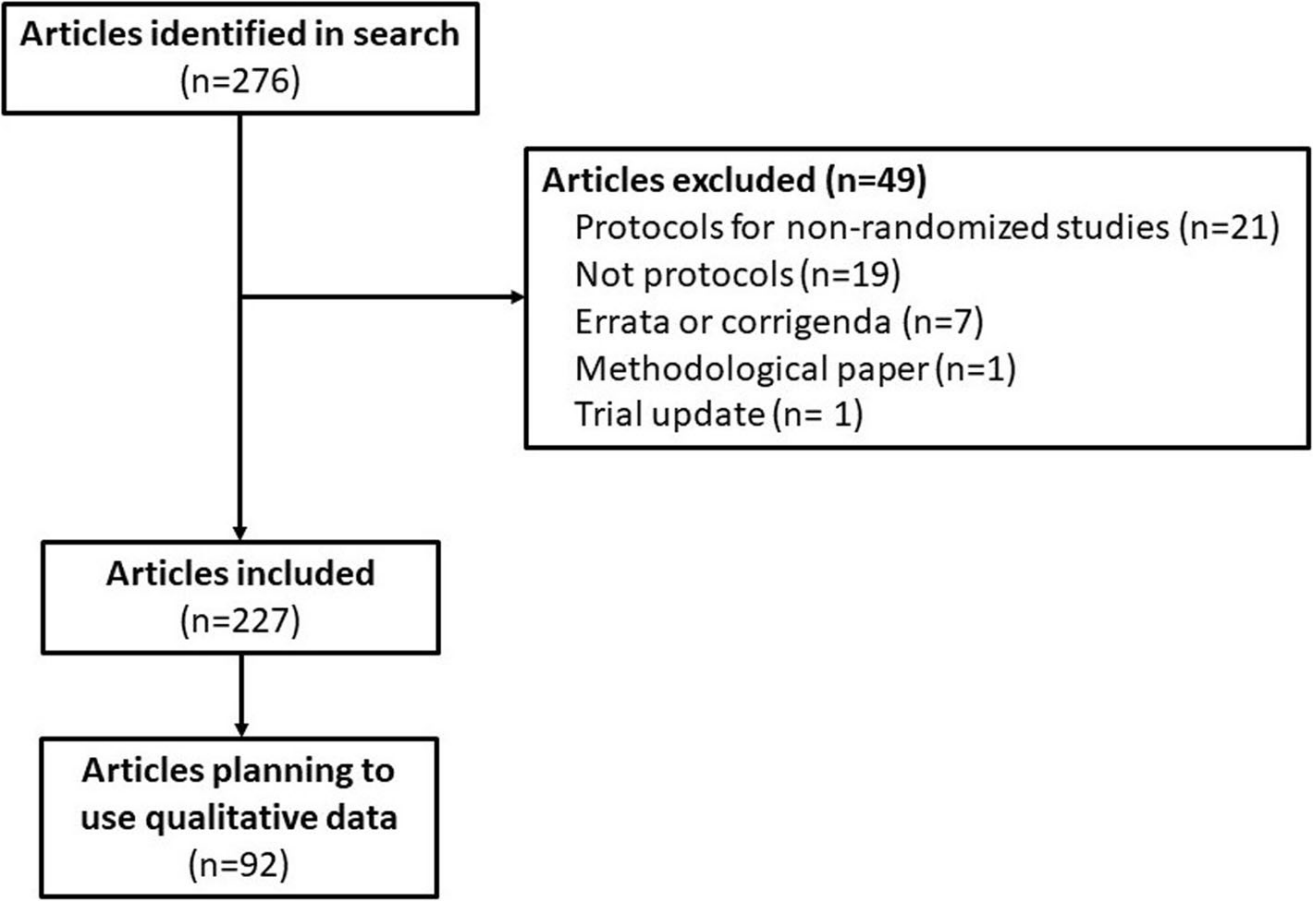
Flow of studies

Our search retrieved 276 articles, of which 49 were not eligible (21 were protocols for non-randomized studies; 19 were full reports and not a protocol; 7 were errata or corrigenda; 1 was a methodological paper; 1 was a trial update). Of the 227 included pilot trial protocols, only 92 (40.5%; 95% CI 34.1–47.2) reported plans to collect qualitative data in their pilot trials. Approximately half (50.2 %) of the included articles were published in *Trials* and conducted in Europe (52.9%). Many of the pilot trials (75.5%) were supported by government or private funding. More pilot trial protocols were published in 2017 than in any other year (31.7%), and the most common intervention type was non-pharmacological (84.6%). The flow of studies is reported in Fig. 1. The complete description of the study characteristics and frequency of qualitative data collection is reported in Table 1.

**Table 1:** Characteristics of included studies

Variable	Use of qualitative data, ***n*** (%)		Total, ***n*** (%), 227 (100)
	Yes, 92 (40.5)	No, 135 (59.5)	
**Journal,** ***n*** **(%)**			
PAFS	23 (25.0)	19 (14.1)	42 (18.5)
BMJ Open	27 (29.3)	44 (32.6)	71 (31.3)
Trials	42 (45.7)	72 (53.3)	114 (50.2)
**Year of publication,** ***n*** **(%)**			
2013	13 (14.1)	21 (15.6)	34 (15.0)
2014	13 (14.1)	24 (17.8)	37 (16.3)
2015	16 (17.4)	18 (13.3)	34 (15.0)
2016	21 (22.8)	29 (21.5)	50 (22.0)
2017	29 (31.5)	43 (31.9)	72 (31.7)
**Region,** ***n*** **(%)**			
North America	15 (16.3)	28 (20.7)	43 (18.9)
Europe	63 (68.5)	57 (42.2)	120 (52.9)
Rest of the world	14 (15.2)	50 (37.0)	64 (28.2)
**Funding,** ***n*** **(%)**			
Industry	25 (28.4)	27 (21.8)	52 (24.5)
Government or private	63 (71.6)	97 (78.2)	160 (75.5)
**Intervention type,** ***n*** **(%)**			
Pharmacological	5 (5.4)	30 (22.2)	35 (15.4)
Non-pharmacological	83 (94.6)	105 (77.8)	192 (84.6)
**Feasibility outcomes (yes),** ***n*** **(%)**	54 (58.7)	69 (51.1)	123 (54.2)
**Sample size reported (yes),** ***n*** **(%)**	91 (98.9)	129 (95.6)	220 (96.9)
**Sample size,** ***n*** **(%)**			
Small (*n* < 60)	45 (49.5)	98 (75.4)	143 (64.7)
Large (*n* ≥ 60)	46 (50.5)	32 (24.6)	78 (35.3)
**Sample size justification,** ***n*** **(%)**^ǂ^			
Adequate	48 (52.7)	51 (39.2)	99 (44.8)
Inadequate	43 (47.3)	79 (60.8)	122 (55.2)
**Study participants interviewed**			
Participants	33 (35.9)	–	33 (35.9)
Investigators	1 (1.1)	–	1 (1.1)
Staff	15 (16.3)	–	15 (16.3)
Others*	12 (13.0)	–	12 (13.0)

*PAFS* Pilot and Feasibility Studies, *BMJ* British Medical Journal

*Caregivers, family members, primary physicians

^ǂ^The minimum study sample size was 6, and the maximum was 7500

After adjusting for covariates, we found that pilot trials that were large (sample size > 60; aOR 2.77; 95% CI 1.47–5.23; *p* = 0.002) and conducted in Europe (aOR 3.86; 95% CI 1.68–8.88; *p* = 0.001) were more likely to plan to collect qualitative data. Pilot trials using pharmacological interventions (aOR 0.20; CI 0.07–0.58; *p* = 0.003) were less likely to plan to collect qualitative data. Our complete univariate and multivariate analyses of factors associated with the collection of qualitative data are presented in Table 2.

**Table 2:** Factors associated with collecting qualitative data^+^

Variable	Crude OR (95%CI)	***p*** value	Adjusted OR (95% CI)	***p*** value
**Journal**				
PAFS	1		1	
BMJ Open	0.45 (0.21–0.99)	0.047	0.80 (0.29–2.26)	0.677
Trials	0.48 (0.24–0.99)	0.046	0.55 (0.20–1.51)	0.244
**Year of publication**	1.02 (0.85–1.23)	0.834	0.97 (0.76–1.24)	0.827
**Region**				
Rest of the world	1		1	
North America	1.91 (0.81–4.53)	0.141	2.19 (0.80–5.99)	0.125
Europe	3.76 (1.88–7.53)	< 0.001	3.86 (1.68–8.88)	0.001
**Funding**				
Industry	1		1	
Government or private	0.67 (0.36–1.26)	0.211	0.81 (0.36–1.84)	0.621
**Intervention type**				
Non-pharmacological	1		1	
Pharmacological	0.21 (0.08–0.56)	0.002	0.20 (0.07–0.58)	0.003
**Sample size**				
Small (*n* < 60)	1		1	
Large (*n* ≥ 60)	3.06 (1.72–5.46)	< 0.001	2.77 (1.47–5.23)	0.002

*CI* confidence interval, *PAFS* Pilot and Feasibility Studies, *BMJ* Open British Medical Journal Open, *OR* odds ratio

^+^The Hosmer and Lemeshow test for model fit indicated good fit (*p* = 0.265)

## Discussion

We found that qualitative data collection is planned for in less than half of the protocols of pilot trials. However, we found that qualitative data are more likely to be collected in pilot trials conducted in Europe, in studies assessing non-pharmacological interventions, and in larger pilot trials. When qualitative data collection was planned for, it was most often collected from study participants.

Our estimate of the frequency with which researchers plan to collect qualitative data may not be an accurate representation of the frequency of use of qualitative methods used in pilot trials because the methods outlined in protocols may not be implemented. Conversely, unplanned methods may be implemented. Despite that, our results show a discrepancy between researchers’ intent to perform qualitative methods and MRC recommendations to use qualitative and quantitative methods concurrently for all pilot studies [5]. It is not surprising that less than half of pilot trial protocols report plans to use qualitative data given the misconceptions about the value of qualitative methods among some researchers, lack of guidance on how to incorporate qualitative research in pilot trials, or concerns with the practicality, reliability, and generalizability of qualitative data [9]. Further research could access researchers’ perspectives on the use of qualitative data in pilot trials. Also, if researchers unduly place emphasis on effectiveness (rather than feasibility) in pilot studies, they may be less likely to use qualitative data. However, the percentage of pilot trials that did plan to use qualitative data in this study was higher than a 2013 study of the use of qualitative methods before a full trial [8]. This suggests that the frequency of qualitative data collection may be on the rise, and current guidance on pilot studies is correcting researchers’ misconceptions about qualitative methods. We strongly encourage researchers to refer to relevant guidance and consider qualitative methods more often for their own pilot studies [11].

It is unclear why studies from Europe were more likely to use qualitative data. However, other studies have found that there are regional differences in reporting quality [20–22], with at least one suggesting that reporting is better in studies from Europe [23]. It is possible that launch of the journal *Pilot and Feasibility Studies* (based in the UK) in 2015 and subsequent publication of guidance for reporting randomized and feasibility trials in the journal (e.g. Consolidated Standards of Reporting Trials statement for reporting feasibility studies) have had the greatest impact in Europe [12–14]. Otherwise, we are not aware of any stringent practices in Europe for pilot trial protocol reporting by agencies or institutions that support studies.

Pilot studies of non-pharmacological interventions were more likely to use qualitative data. This may be because non-pharmacological interventions are more amenable to modifications based on user feedback. For example, a 2016 mixed methods feasibility and pilot cluster randomized controlled trial examined the use of a nurse-led goal-setting intervention for adults with asthma using semi-structured interviews [24]. They identified several issues with the intervention, including the need to make it more team-based and simplistic. They also identified a variety of procedural issues (e.g. reducing the impact of the intervention on consultation time). In comparison, it may not be possible to change as many aspects of pill-based, pharmaceutical interventions. Even though qualitative data would still have value (e.g. patient feedback may help to inform the design of pills—two smaller tablets vs. one—or help in adapting language during recruitment if patients report symptoms or issues that impact their swallowing of oral pills), its applicability may be limited. However, even if the intervention may not be amenable to change, there is value in using qualitative methods to assess acceptability [25, 26].

Additionally, research indicates that larger studies tend to be better reported [16], although this may not be applicable to pilot studies given their generally smaller sample sizes. This is likely due to the availability of resources for larger studies, which may include larger multidisciplinary teams and funds for better design and more in-depth data collection.

Not surprisingly, investigators most often planned to collect qualitative data from study participants. Participant interviews may provide rich data on satisfaction with the intervention, acceptability of the procedures, and issues that may prevent participants from enrolling in the main trial. Family and caregivers may provide such information as well. Likewise, staff working in the hospitals in which the study will be conducted can provide information on how the study affects their workflow as well as any logistic challenges that they might face. Careful consideration of who to interview can optimize the chances of success in the future trial. For example, in a pilot trial of a structured physical activity intervention for colorectal cancer patients, Hubbard et al. identified recruitment biases, imprecision, and issues with adherence by interviewing clinicians and patients [27, 28].

This study is not without some limitations. Our search for pilot trial protocols was focused on only 3 leading journals from 2013 to 2017. There may be other journals that publish protocols for pilot studies of which are unaware. Therefore, it is possible that our data may not be representative of patterns of plans to use qualitative data in other journals. However, the strengths of this study are robust methods used to identify, screen, and analyse the data, and the novelty of the findings.

## Conclusion

Qualitative data is not incorporated in the designs of pilot studies often enough, despite its potential value in informing intervention development and the definitive study design. Investigators conducting pilot studies are encouraged to consider the role of qualitative data in their feasibility assessments, particularly when planning the pilot trial.

## Data Availability

The full dataset is available from the corresponding author upon request.

## Abbreviations

aOR: Adjusted odds ratio
BMJ: *British Medical Journal*
CI: Confidence interval
OR: Odds ratio
PAFS: *Pilot and Feasibility Studies*
